# *MDM2* promoter SNP55 (rs2870820) affects risk of colon cancer but not breast-, lung-, or prostate cancer

**DOI:** 10.1038/srep33153

**Published:** 2016-09-14

**Authors:** Reham Helwa, Liv B. Gansmo, Pål Romundstad, Kristian Hveem, Lars Vatten, Bríd M. Ryan, Curtis C. Harris, Per E. Lønning, Stian Knappskog

**Affiliations:** 1 Section of Oncology, Department of Clinical Science, University of Bergen, 5020 Bergen, Norway; 2 Department of Oncology, Haukeland University Hospital, 5021 Bergen, Norway; 3 Department of Public Health, Faculty of Medicine, Norwegian University of Science and Technology, 7489 Trondheim, Norway; 4 Laboratory of Human Carcinogenesis, Center for Cancer Research, National Cancer Institute, Bethesda, MD, 20892, USA

## Abstract

Two functional SNPs (SNP285G > C; rs117039649 and SNP309T > G; rs2279744) have previously been reported to modulate Sp1 transcription factor binding to the promoter of the proto-oncogene *MDM2*, and to influence cancer risk. Recently, a third SNP (SNP55C > T; rs2870820) was also reported to affect Sp1 binding and *MDM2* transcription. In this large population based case-control study, we genotyped *MDM2* SNP55 in 10,779 Caucasian individuals, previously genotyped for SNP309 and SNP285, including cases of colon (n = 1,524), lung (n = 1,323), breast (n = 1,709) and prostate cancer (n = 2,488) and 3,735 non-cancer controls, as well as 299 healthy African-Americans. Applying the dominant model, we found an elevated risk of colon cancer among individuals harbouring SNP55TT/CT genotypes compared to the SNP55CC genotype (OR = 1.15; 95% CI = 1.01–1.30). The risk was found to be highest for left-sided colon cancer (OR = 1.21; 95% CI = 1.00–1.45) and among females (OR = 1.32; 95% CI = 1.01–1.74). Assessing combined genotypes, we found the highest risk of colon cancer among individuals harbouring the SNP55TT or CT together with the SNP309TG genotype (OR = 1.21; 95% CI = 1.00–1.46). Supporting the conclusions from the risk estimates, we found colon cancer cases carrying the SNP55TT/CT genotypes to be diagnosed at younger age as compared to SNP55CC (*p* = 0.053), in particular among patients carrying the SNP309TG/TT genotypes (*p* = 0.009).

The *MDM2* gene is a proto-oncogene executing its main oncogenic effects by ubiquitin ligase activity, targeting p53 for proteosomal degradation^1^,^2^. *MDM2* has been reported to be overexpressed via several different molecular mechanisms in many cancer forms. In addition to gene amplifications, the *MDM2* gene is also over-expressed by increased transcription as well as enhanced translation^3^,^4^. *MDM2* expression is regulated via two promoters^5^,^6^ and these mediate expression of two different transcripts with distinct first exons. However, since the translation initiation site is located in the third exon, the open reading frame remains the same.

A large number of SNPs within the promoter regions of *MDM2* have been identified, but only a handful of these have been functionally characterized and assessed for potential associations with cancer^7^,^8^,^9^,^10^. More than a decade ago, Bond and colleagues identified the SNP309G-allele (rs2279744) to elongate a binding site for the Sp1 transcription factor, causing increased *MDM2* expression and elevated cancer risk^8^. Subsequent case-control studies, however, have generated conflicting results in Caucasian populations, but in general linked the G-allele to increased cancer risk in Asian populations^11^,^12^. Later, we and others identified a second polymorphism (SNP285G > C; rs117039649), located in close proximity (24 bp upstream) to SNP309^9^,^13^. SNP285 is also located within a Sp1 binding site. Contrasting the SNP309 G-allele, the minor allele (C) of SNP285 reduced Sp1 binding, thereby leading to reduced *MDM2* expression^9^. Further, SNP285C was associated with a reduced risk of cancer of the breast, ovary and endometrium^9^,^14^. Notably, the SNP285C-allele has been observed only in Caucasians and some neighbouring populations and is absent in Sub-Saharan Africans and Eastern Asian populations^15^, a finding providing a possible explanation for the observed differences in impact of SNP309 in Caucasian and Asian studies.

Recently, a third SNP affecting Sp1 binding to the *MDM2* promoter P2 (SNP55C > T; rs2870820) was reported (Fig. 1). Here, the minor T-allele was found to enhance Sp1 binding and thereby increase *MDM2* expression^16^. However, with the exception of the original paper assessing SNP55 genotype distribution across 45 endometrial cancer patients and an equally sized control group^16^, no formal assessment of the potential link between this SNP and cancer risk has been made.

In the present study, we genotyped *MDM2* SNP55 across a large population based study and assessed potential correlations to incidental cancer risk by comparing the genotype status between individuals diagnosed with cancer versus controls. Further, we genotyped SNP55 in healthy African-Americans for ethnic comparison of genotype frequencies and haplotype structures with other *MDM2* promoter SNPs.

## Results

### Distribution of *MDM2* SNP55

**Figure 2:**
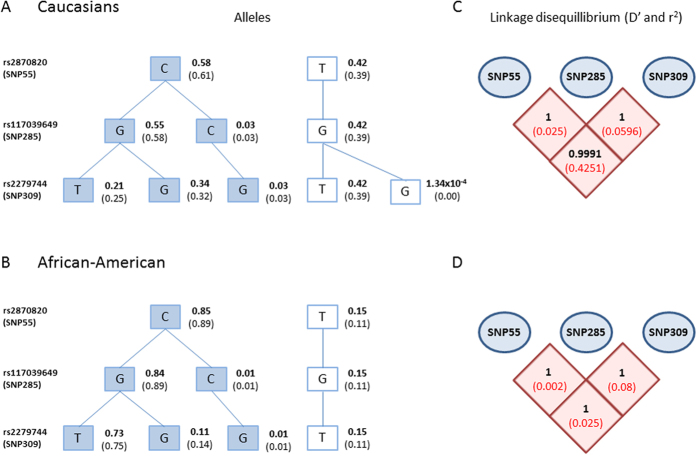
(**A**) Haplotype-tree based on *MDM2* SNP55, 285, and 309. The tree and frequencies are based on 3,725 healthy individual included in this study (written in bold) and 503 Caucasian individuals from 1000 genome project (frequencies in brackets). (**B**) The tree and frequencies are based on 299 healthy individuals of African-American ethnicity (written in bold) and 61 African-Americans from the 1000 genome project (frequencies in brackets). (**C**) Pairwise linkage disequilibrium (LD) between *MDM2* SNP55, 285, and 309. D′ (in bold) and r^2^ (in red) were calculated among the Caucasian healthy controls in the present study. (**D**) LD between SNP55, 285, and 309 among the 299 African-Americans; D′ (in bold) and r^2^ (in red).

In the present study, we successfully genotyped the *MDM2* SNP55C>T (rs2870820) in a total of 10,751 Caucasian (Norwegian) individuals (3,725 healthy controls and 7,026 cancer cases) as well as 299 healthy African-Americans (for ethnic comparison). Among the healthy Caucasians, the minor allele frequency (MAF) was found to be 0.42 (Fig. 2A). These results are in line with data extracted from the Caucasian subset of the 1000 genome project (www.1000genomes.org), where the MAF is 0.39 (Fig. 2A). The genotype distribution in the Caucasian controls (Table 1) was found to be in Hardy-Weinberg equilibrium (*p* > 0.05). Interestingly, the distribution of SNP55 was different in the African-American cohort, where the MAF was 0.15 (Fig. 2B). Similarly to our observations for Caucasians, these results were in line with the 1000 genome project (MAF for the African-American subset: 0.11; Fig. 2B).

For all Caucasian cases and controls in the present study, we have previously reported the genotype status for the two functional *MDM2* promoter SNPs; 285 (rs117039649) and 309 (rs2279744)^17^. Among the healthy controls, we observed a strong linkage disequilibrium between SNP55 and SNP309 (D′ = 0.9991; r^2^ = 0.425; Fig. 2C). We observed no individuals harbouring the minor alleles of SNP55 and SNP285 concomitantly. Thus, SNP55 was found to be in complete linkage disequilibrium with SNP285, with the SNP55T-allele being linked to the SNP285G-allele (D′ = 1; r^2^ = 0.025; Fig. 2C). These observations were confirmed in the Caucasian sub set of the 1000 genome project, where SNP55 was in complete linkage disequilibrium with both SNP285 (D′ = 1; r^2^ = 0.02) and SNP309 (D′ = 1; r^2^ = 0.357). We found a very strong linkage disequilibrium between SNP55 and SNP285 – SNP309 also to be the case within the African-American sample (D′ = 1; r^2^ = 0.002 and D′ = 1; r^2^ = 0.025, respectively; Fig. 2D).

### Influence of *MDM2* SNP55 genotype on cancer risk

**Figure 3:**
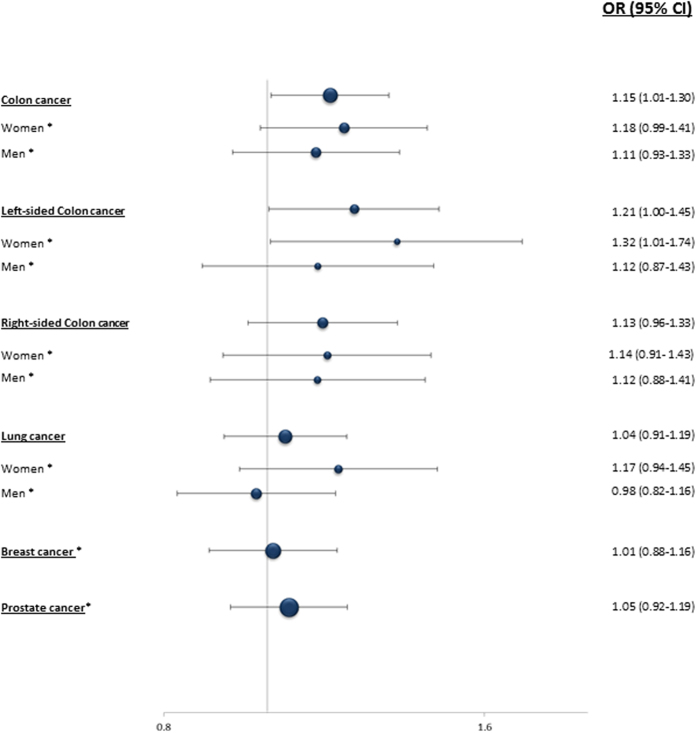
Impact of *MDM2* SNP55 on cancer risk. Forest plot illustrating the effect of SNP55 (dominant model) on the risk of colon-, lung-, breast-, and prostate cancer compared to healthy control. ORs for breast and prostate cancer were calculated compared to gender matched controls (marked with a *).

Applying the dominant model for the minor allele (*MDM2* SNP55TT + TC versus CC), no significant effect on risk was found for lung- (OR = 1.04; 95% CI = 0.91–1.19), breast (OR = 1.01; 95% CI = 0.88–1.16), or prostate cancer (OR = 1.05; 95% CI = 0.92–1.19; Table 1; Fig. 3). However, we observed an increased risk of colon cancer (OR = 1.15; 95% CI = 1.01–1.30).

Stratifying the colorectal cancer cases according to tumor site (left versus right sided cancer), we found a significant increase in the risk of left sided cancer (OR = 1.21; 95% CI = 1.00–1.45) but not for right sided cancers. Furthermore, by stratifying the left sided cases by gender, a relatively high increase in risk of left colon cancer was observed among females (OR = 1.32; 95% CI = 1.01–1.74), but not for males (Fig. 3). Although similar results were observed under the additive model (Supplementary Table S1), notably, the increased risk was strongest linked to heterozygosity for the T-allele: Applying the co-dominant model we found the OR for colon cancer in general to be 1.17 (CI = 1.02–1.34) and the OR for left sided cancer among females to be 1.39 (CI = 1.05–1.85; Supplementary Table S2).

In contrast, applying the recessive model (*MDM2* SNP55TT versus TC+CC), no significant effect of SNP55 status was found for any of the four investigated cancer types (Table 1).

### Influence of *MDM2* SNP55 genotype on age at cancer onset

**Figure 4:**
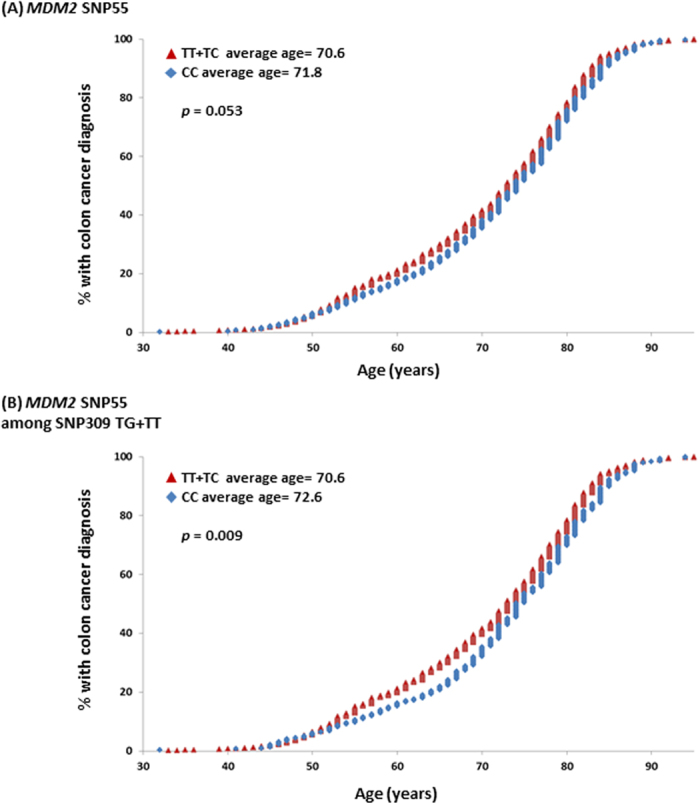
Impact of *MDM2* SNP55 and 309 on age at colon cancer diagnosis. (**A**) Cumulative percentage of individuals with colon cancer diagnosis as a function of age, among SNP55 T-allele carriers (TT and TC genotypes combined; red triangles) compared to non-T-allele carriers (CC genotype; blue diamonds). (**B**) Cumulative percentage of individuals with colon cancer diagnosis as a function of age, among SNP55 T-allele carriers (TT and TC genotypes combined; red triangles) compared to non-T-allele carriers (CC genotype; blue diamonds), restricted to individuals harbouring the *MDM2* SNP309 T-allele (TG and TT genotypes).

Given that germline cancer risk factors often are linked to a younger age at diagnosis, we compared mean age at diagnosis for individuals carrying the different *MDM2* SNP55 genotypes within each diagnostic group. For the lung-, breast-. and prostate cancer cases, we found no effect of SNP55 status on age at diagnosis. However, among patients diagnosed with colon cancer, the mean age at diagnosis was 70.6 years for individuals harbouring the SNP55 TT/TC genotypes but 71.8 years for those harbouring the SNP55 CC genotype (dominant model; *p* = 0.053; Table 2; Fig. 4A).

### *MDM2* SNP55 and cancer risk among individuals carrying the SNP309T-allele

Since the minor SNP55-allele is limited to the SNP55T/309T haplotype, we assessed the cancer risk related to SNP55 status within the subgroups of individuals harboring either the SNP309TT or the TG genotype. Applying the dominant model, we found the SNP55 T-allele to be associated with increased risk of colon cancer among individuals with the SNP309TG genotype (OR = 1.21; 95% CI = 1.00–1.46; Supplementary Table S3), but not among individuals harbouring the SNP309TT genotype, possibly indicating a SNP309-allele to be present in order for SNP55T to confer cancer risk.

Supporting this observation, in the subgroup of individuals harbouring the SNP309TT or TG genotypes, we found SNP55T-allele carriers (individuals harbouring the TT or the TC genotypes) to have a lower age at colon cancer diagnosis as compared to CC-carriers (average age at diagnosis 70.6 versus 72.6 years, respectively; *p* = 0.009; Table 2; Fig. 4B).

### *MDM2* promoter haplotypes and cancer risk

In addition to assessing the OR for individuals with the different genotypes, we also estimated ORs for the four observed haplotypes across the three SNPs 55, 285 and 309 (Fig. 2A). Although not reaching statistical significance we found the allele carrying SNP55T (SNP55T, 285G, 309T) to be associated with the highest risk of colon cancer (OR = 1.09; CI = 0.99–1.21, compared to the lowest risk haplotype SNP55C, 285G, 309G; Supplementary Table S4). For the remaining three cancer forms, no association between haplotypes and cancer risk was observed.

### *MDM2* SNP55 and *MDM2* expression

Based on our findings above, we assessed whether the SNP55 status affected MDM2 expression more in healthy colon tissue than in the healthy tissues of the three other organs included in the present study. Thus, we extracted eQTL data from the GTEx Portal (www.gtexportal.org). Although heterozygosity for SNP55 seemed to lead to increased levels of *MDM2* expression in healthy colon tissue, the effect of the T-allele was not of statistical significance. Among the four tissues, prostate was the tissue where the T-allele seemed to have the largest impact in terms of increased *MDM2* expression (p = 0.075; Supplementary Figure S1).

## Discussion

Recently, SNP55 (rs2870820) was identified as a functional polymorphism in the P2 promoter of the *MDM2* proto-oncogene. While this variant modulates the binding affinity between the transcription factors Sp1 and NFkB and the *MDM2* promoter, and elevates *MDM2* transcription^16^, the only previous study assessing its potential effect on cancer risk included 45 patients diagnosed with endometrial cancer and a similar number of controls^16^.

In addition to SNP55, two other SNPs in *MDM2* P2 promoter (SNP285 and 309) have previously been reported to influence Sp1 binding sites and cancer risk^9^,^14^,^15^,^17^. Thus, in the present study, the risk of *MDM2* SNP55 was assessed in a large population based study of Norwegian incident cancer cases and controls, where SNP285 and SNP309 status had been determined previously. Contrasting previous findings for SNP285 and 309 in the Norwegian population^17^, SNP55-status was found to affect the risk of colon cancer.

Germline mutations are often associated with early onset of cancer. For instance, for several cancer predisposing syndromes, the cancer risk is age dependent^18^,^19^, and it is well established that germline mutations of *BRCA1* and *BRCA2* are associated with early cancer onset^20^,^21^. Thus, we sought to validate our findings from the cases-control designed calculations by comparing the age at cancer diagnosis between individuals harbouring the different SNP55 genotypes, within each cancer type. Importantly, our observation of an increased risk of colon cancer linked to the SNP55T-allele was supported by the finding of a younger age at diagnosis among colon cancer patients harbouring this allele.

The two sides of the human colon differ in origin; the right colon (proximal) originates from the embryonic midgut, whereas the left colon (distal) develops from hindgut. Also, the genetic mechanisms behind the carcinogenesis process are disparate^22^. Much evidence indicates that colorectal cancers initiate with site preference. For instance, hereditary nonpolyposis colon cancer (HNPCC) related to germline mutations in the DNA mismatch genes^23^,^24^,^25^ in general locates to the right side of the colon^26^, while the tumorigenesis process of familial adenomatous polyposis (FAP) is dominant in the left-sided colon cancers^27^. Given this background we stratified our analyses and assessed the impact of SNP55 status in left sided and right sided colon cancer separately. Interestingly, our results revealed an increased risk of left-sided but not right-sided colon cancer with respect to *MDM2* SNP55 status.

*MDM2* SNP55, similarly to SNP309 and SNP285 may influence *MDM2* transcription by modulating Sp1 binding. However, these three SNPs influence the risk of cancer in different organ systems. While the reason for this is unknown, notably SNP55 also affects the binding of NFkB^16^ and SNP285 resides in a Sp1 binding site overlapping with an estrogen receptor (ER) half-site^14^. Thus, one may speculate the observed differences with respect to cancer risk could be due to interactions with other tissue-specific transcription factors.

In the present study, although reduced samples sizes caused somewhat wide CIs when stratifying for gender, SNP55 status was also found to affect the risk for left sided colon cancer in a gender-specific manner. A gender specific effect of *MDM2* SN309 was previously reported in lung cancer^17^. While the cancer risk reducing effects of SNP285C so far has been limited to female cancers only (breast, ovarian and endometrial but not prostate or lung cancers)^9^,^14^,^17^,^28^, no gender specific effect of SNP285 in colon cancer was observed^17^. Other studies have linked the SNP309G-allele to high risk of colorectal cancer specifically in premenopausal women^29^,^30^. Further, women harbouring SNP309G have an increased risk of developing B-cell lymphoma, melanoma, and lung cancers^30^,^31^,^32^, contrasting no effects on risk for the same cancers in males. These findings have been suggested to be caused by proximity between estrogen response elements (ERE) and Sp1 binding sites which increase the binding affinity co-operatively and subsequently *MDM2* expression^14^,^33^,^34^. Regarding SNP55, this polymorphism is located further away from known EREs and it is less likely that cooperativity between Sp1 and ER may explain the gender specific effects of SNP55.

In summary, we found individuals harbouring the SNP55TT or CT genotype to have a significantly increased risk of colon cancer, in particular females and left-sided colon cancer. These findings were corroborated by a younger age at colon cancer diagnosis among patients carrying the SNP55 T-allele.

## Material and Methods

### Study population

In this case-control study, samples from cancer cases and healthy controls were obtained from the population-based cohort of Norway (CONOR) study^35^. Incident cancers were identified by linking individuals’ identity in the CONOR study to the Norwegian cancer registry (from entry to the end of 2010). Thus, we included 7,044 cancer cases (1,524 colon cancers, 1,323 lung cancers, 1,709 breast cancers, and 2,488 prostate cancers). From the same cohort, we also analysed as sample of 3,735 healthy controls that were matched to the cases with respect to age (same fraction of individuals in five years groups), giving a total samples size of 10,779 individuals. For technical reasons, 28 samples were excluded. Therefore, the presented results in the present study includes the genotypes of 10,751 individuals (3,725 non-case healthy controls, 1,515 colon cancers, 1,321 lung cancers, 1,707 breast cancers, and 2,483 prostate cancers).

The samples included in this study have previously been genotyped for the *MDM2* SNPs SNP285 (rs117039649) and SNP309 (rs2279744)^17^.

In addition, to assess ethnic differences, a sample set of healthy African-Americans (n = 299) was investigated. These samples were from Laboratory of Human Carcinogenesis, Center for Cancer Research, National Cancer Institute Bethesda, USA. The African-American samples were a part of case control study described previously^28^,^36^. The population controls from this cohort were identified from the Department of Motor Vehicles, MD, USA and frequency matched to cases according to age and gender.

### Ethics

All experiments were carried out in accordance to the national guidelines (Norway and U.S.) for research on human material. All samples donors have provided written informed consent to anonymous genetic testing for research purpose. The Norwegian part of the study is approved by the Regional Committee for Ethics in Medical Research (REK Midt-Norge), while the American part was approved by the National Cancer Institute, the VA Medical System and the University of Maryland Medical System.

### *MDM2* promoter SNP55 screening

*MDM2* SNP55 (rs2870820) was genotyped in all samples using custom LightSNiP assays (TIB MOLBIOL Syntheslabor GmbH, Berlin, Germany) on a LightCycler 480 II instrument (Roche, Basel, Switzerland) as previously described^10^. The amplifications were performed in a total reaction volume of 10 μl, containing 1 μl LightCycler^®^ FastStart DNA Master HybProbe mix (Roche diagnostic), 0.25 μl LightSNiP mix (TIB MOLBIOL), 3 mM MgCl_2,_ and 10–50 ng DNA. Thermal cycling conditions were: 10 minutes initial denaturation, followed by 45 cycles of 10 seconds denaturation at 95 °C, 10 annealing seconds at 60 °C, and 15 seconds elongation at 72 °C. Then, high resolution melting (HRM) was performed as follows: initial denaturation at 95 °C for 30 seconds, followed by melting from 40 °C to 75 °C with a ramp rate of 0.19 °C/sec and a final cooling at 40 °C for 30 seconds. Subsequently, the HRM curves were analysed using Melt Curve Genotyping module in the LightCycler 480 software version 1.5.

### Statistical analysis

All statistical analyses were performed using SPSS software statistics package (version 22). Potential associations of *MDM2* SNP55 and cancer risk for colon, lung, breast, and prostate cancers were assessed by odds ratio (OR). Odds ratios are given with 95% confidence intervals (CI). Differences in age at cancer onset between individuals with the different genotypes were assessed using Kruskal-Wallis rank test for comparison of three groups and Mann-Whitney rank test for comparison of two groups. Potential deviations from Hardy-Weinberg equilibrium were assessed by calculating the expected genotype distribution based on the observed allele frequencies and compared to the observed genotype distribution, using Chi-square test. All p-values given are two sided.

### Linkage disequilibrium assessments

The linkage disequilibrium between SNP pairs was calculated using the equation: *D* = x11− p1q1, where x11 is the frequency of the haplotype, p1 is frequency of the first SNP, and q1 is frequency of the second SNP. D′ was calculated using the equation: D′ = D/D_max_, where, if D > 0: D_max_ = min p1q2 or p2q1 and if D < 0: D_max_ = min p1q1 or p2q2. Also, r^2^ was calculated using the equation r^2^ = D^2^/p1p2q1q2. D′ and r^2^ were calculated separately among healthy individuals of the two ethnic groups (n = 3,725 Norwegians and n = 299 African-Americans).

## Additional Information

**How to cite this article**: Helwa, R. *et al. MDM2* promoter SNP55 (rs2870820) affects risk of colon cancer but not breast-, lung-, or prostate cancer. *Sci. Rep.* **6**, 33153; doi: 10.1038/srep33153 (2016).

## Supplementary Material

Supplementary Information

## Figures and Tables

**Figure 1:**
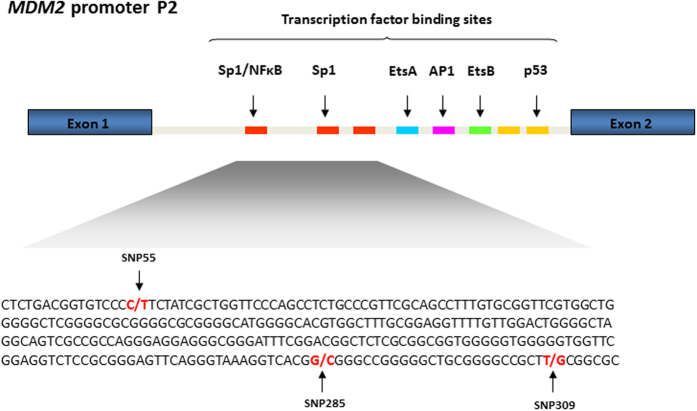
*MDM2* promoter P2. The promoter (P2) is located between exons 1 and 2 and regulated by many transcription factors including Sp1. Part of the promoter P2 sequence is shown in detail, in order to illustrate the sequence context of SNP55 and the two other SNPs affecting Sp1 binding (SNP285 and SNP309).

**Table 1:** *MDM2* SNP55 distribution and risk estimates for colon, lung, breast, and prostate cancer.

	Genotype *n* (%)			OR (95% CI)	*p*-value	OR (95% CI)	*p*-value
	CC	CT	TT	CT+TT vs CC		TT vs CC+CT	
Controls	1285 (34.5)	1763 (47.3)	677 (18.2)	1.00	—	1.00	—
Women^1^	638 (34.3)	871 (46.9)	349 (18.8)	1.00	—	1.00	—
Men^2^	647 (34.7)	892 (47.8)	328 (17.6)	1.00	—	1.00	—
Colon cancer	477 (31.5)	764 (50.4)	274 (18.1)	1.15 (1.01–1.30)	0.039	0.99 (0.85–1.16)	0.968
Women^1^	237 (30.7)	389 (50.4)	146 (18.9)	1.18 (0.99–1.41)	0.076	1.01 (0.81–1.25)	0.956
Men^2^	240 (32.3)	375 (50.5)	128 (17.2)	1.11 (0.93–1.33)	0.272	0.98 (0.78–1.22)	0.864
Left Colon cancer	189 (30.4)	326 (52.4)	107 (17.2)	1.21 (1.00–1.45)	0.049	0.94 (0.75–1.17)	0.612
Women^1^	83 (28.3)	158 (53.9)	52 (17.8)	1.32 (1.01–1.74)	0.046	0.93 (0.68–1.29)	0.747
Men^2^	106 (32.2)	168 (51.1)	55 (16.7)	1.12 (0.87–1.43)	0.413	0.94 (0.69–1.29)	0.753
Right Colon cancer	261 (31.8)	403 (49.2)	156 (19)	1.13 (0.96–1.33)	0.154	1.06 (0.87–1.28)	0.583
Women^1^	135 (31.5)	209 (48.7)	85 (19.8)	1.14 (0.91–1.43)	0.282	1.07 (0.82–1.39)	0.633
Men^2^	126 (32.2)	194 (49.6)	71 (18.2)	1.12 (0.88–1.41)	0.380	1.04 (0.78–1.38)	0.771
Lung cancer	444 (33.6)	620 (46.9)	257 (19.5)	1.04 (0.91–1.19)	0.566	1.09 (0.93–1.28)	0.303
Women^1^	152 (31.0)	245 (49.9)	94 (19.1)	1.17 (0.94–1.45)	0.163	1.02 (0.80–1.32)	0.846
Men^2^	292 (35.2)	375 (45.2)	163 (19.6)	0.98 (0.82–1.16)	0.793	1.15 (0.93–1.41)	0.214
Breast cancer^1^	581(34.0)	804 (47.1)	322 (18.9)	1.01 (0.88–1.16)	0.860	1.01 (0.85–1.19)	0.966
Prostate cancer^2^	834 (33.6)	1227 (49.4)	422 (17.0)	1.05 (0.92–1.19)	0.477	0.96 (0.82–1.13)	0.627

^1^Calculations against female healthy controls.

^2^Calculations against male healthy controls.

**Table 2:** Age at cancer diagnosis among the different genotypes of SNP55.

	Average age at cancer diagnosis (dominant model)			Average age at cancer diagnosis (recessive model)		
	CC	CT+TT	*p-*value	CC+CT	TT	*p-*value
SNP55						
Colorectal	71.80	70.60	0.053	71.03	70.8	0.645
Lung	70.49	69.61	0.180	69.89	69.97	0.948
Breast	59.82	60.59	0.364	60.24	60.7	0.577
Prostate	72.00	71.52	0.167	71.66	71.80	0.715
SNP55 among SNP309 TG and TT						
Colorectal	72.56	70.6	0.009	71.09	70.8	0.583
Lung cancer	70.03	69.61	0.579	69.63	69.97	0.718
Breast	59.93	60.59	0.508	60.36	60.70	0.65
Prostate	71.80	71.51	0.472	71.53	71.81	0.512
SNP55 among SNP309 TG						
Colorectal	72.73	70.88	0.032	71.49	na^1^	na
Lung	69.54	70.24	0.410	69.99	na^2^	na
Breast	59.58	60.28	0.518	60.04	na^3^	na
Prostate	71.67	71.52	0.824	71.57	na^3^	na
SNP55 among SNP309 TT						
Colorectal	72.04	70.35	0.301	70.31	70.86	0.657
Lung	71.65	69.13	0.078	68.97	69.94	0.297
Breast	61.35	60.85	0.590	61.08	60.70	0.662
Prostate	72.31	71.50	0.346	71.43	71.81	0.323

^1^The number of samples harbouring TT is two samples.

^2^The number of samples harbouring TT is one sample.

^3^No samples with TT allele.
