# Supplementary material for: MDM2 promoter SNP55 (rs2870820) affects risk of colon cancer but not breast-, lung-, or prostate cancer
Source: Sci Rep. 2016 Sep 14;6:33153. doi: 10.1038/srep33153 (PMC5022009; doi:10.1038/srep33153)
Supplement: Supplementary Information [file srep33153-s1.pdf]

MDM2 promoter SNP55 (rs2870820) affects risk of colon cancer but not breast-, lung-, or prostate cancer

Reham Helwa[1,2,*] Liv B. Gansmo[1,2], Pål Romundstad[3], Kristian Hveem[3], Lars Vatten[3], Bríd M. Ryan[4], Curtis C. Harris[4], Per E. Lønning[1,2] and Stian Knappskog[1,2,‡]

[1]Section of Oncology, Department of Clinical Science, University of Bergen, 5020 Bergen, Norway

[2]Department of Oncology, Haukeland University Hospital, 5021 Bergen, Norway

[3]Department of Public Health, Faculty of Medicine, Norwegian University of Science and Technology, 7489 Trondheim, Norway

[4]Laboratory of Human Carcinogenesis, Center for Cancer Research, National Cancer Institute, Bethesda, MD, 20892, USA.

*Associated affiliation: Department of Zoology, Faculty of Science, Molecular Biology Lab, Ain Shams University, Cairo, Egypt

Keywords: MDM2, polymorphism, SNP55 (rs2870820), SNP285 (rs117039649), SNP309 (rs2279744), cancer risk

‡ Corresponding author

Stian Knappskog

Department of Clinical Science, University of Bergen, 5021 Bergen, Norway

Tel: (+47) 55976447;

Fax: (+47) 55972046;

E-mail: stian.knappskog@uib.no

Supplementary Table S1. *MDM2* SNP55 distribution and risk estimates under the additive model.

	CC	2xTT +CT	odds ratio	CI	p value
Controls	1285	3117	1.00	-	-
Women[1]	638	1569	1.00	-	-
Men[2]	647	1548	1.00	-	-
Colon cancer	477	1312	1.134	1.002-1.283	0.047
Women[1]	237	681	1.168	0.982-1.391	0.080
Men[2]	240	631	1.099	0.923-1.309	0.310
Left Colon cancer	189	540	1.178	0.986-1.407	0.077
Women[1]	83	262	1.284	0.986-1.670	0.072
Men[2]	106	278	1.096	0.861-1.396	0.503
Right Colon cancer	261	715	1.129	0.966-1.320	0.128
Women[1]	135	379	1.142	0.919-1.418	0.254
Men[2]	126	336	1.115	0.891-1.395	0.367
Lung cancer	444	1134	1.053	0.927-1.196	0.437
Women[1]	152	433	1.158	0.942-1.424	0.179
Men[2]	292	701	1.003	0.851-1.183	1.00
Breast cancer[1]	581	1448	1.013	0.887-1.158	0.865
Prostate cancer[2]	834	2071	1.038	0.919-1.173	0.554

Supplementary Table S2: *MDM2* SNP55 distribution and risk estimates for colon, lung, breast and

	Genotype *n* (%)			OR (95% CI)	*p*-value	OR (95% CI)	*p*-value
	**CC**	**CT**	**TT**	**Codominant model TT vs CC**		**Codominant model CT vs CC**	
Controls	1285 (34.5)	1763 (47.3)	677 (18.2)	1.00	-	1.00	-
Women[1]	638 (34.3)	871 (46.9)	349 (18.8)	1.00	-	1.00	-
Men[2]	647 (34.7)	892 (47.8)	328 (17.6)	1.00	-	1.00	-
Colon cancer	477 (31.5)	764 (50.4)	274 (18.1)	0.98 (0.82-1.18)	0.89	1.17 (1.02-1.34)	0.026
Women[1]	237 (30.7)	389 (50.4)	146 (18.9)	1.13 (0.88-1.43)	0.35	1.2 (0.99-1.46)	0.06
Men[2]	240 (32.3)	375 (50.5)	128 (17.2)	1.05 (0.82-1.35)	0.70	1.13 (0.94-1.37)	0.21
Left Colon cancer	189 (30.4)	326 (52.4)	107 (17.2)	1.08 (0.83-1.39)	0.60	1.26 (1.04-1.53)	0.02
Women[1]	83 (28.3)	158 (53.9)	52 (17.8)	1.15 (0-79-1.66)	0.50	1.39 (1.05-1.85)	0.02
Men[2]	106 (32.2)	168 (51.1)	55 (16.7)	1.02 (0.72-1.46)	0.93	1.15 (0.88-1.50)	0.32
Right Colon cancer	261(31.8)	403 (49.2)	156 (19)	1.13 (0.91-1.41)	0.26	1.13 (0.95-1.34)	0.18
Women[1]	135 (31.5)	209 (48.7)	85 (19.8)	1.15 (0.85-1.56)	0.39	1.13 (0.89-1.44)	0.33
Men[2]	126 (32.2)	194 (49.6)	71 (18.2)	1.11 (0.81-1.53)	0.51	1.12 (0.87-1.43)	0.38
Lung cancer	444 (33.6)	620 (46.9)	257 (19.5)	1.10 (0.92-1.32)	0.31	1.02 (0.88-1.17)	0.83
Women[1]	152 (31.0)	245 (49.9)	94 (19.1)	1.13 (0.85-1.51)	0.42	1.18 (0.94-1.48)	0.15
Men[2]	292 (35.2)	375 (45.2)	163 (19.6)	1.10 (0.87-1.39)	0.44	0.93 (0.78-1.12)	0.45
Breast cancer[1]	581(34.0)	804 (47.1)	322 (18.9)	1.01 (0.84-1.22)	0.92	1.01 (0.87-1.18)	0.88
Prostate cancer[2]	834 (33.6)	1227 (49.4)	422 (17.0)	0.99 (0.84-1.19)	1.00	1.07 (0.93-1.22)	0.36

prostate cancer, under the co-dominant model

[1] calculations against female healthy controls.

[2] calculations against male healthy controls.

Supplementary Table S3. *MDM2* SNP55 distribution and cancer risk among within subgroups of *MDM2* SNP309 genotypes

Table S3(A)

**SNP309 TT+TG**	**Genotype *n* (%)**			**OR (95% CI)**	***p*-value**	**OR (95% CI)**	***p*-value**
	**CC**	**CT**	**TT**	**CT+TT vs CC**		**TT vs CC+CT**	
Controls	787 (24.4)	1763 (54.6)	677(21)	1.00	-	1.00	-
Women[1]	385 (24.0)	871 (54.3)	349 (21.7)	1.00	-	1.00	-
Men[2]	402 (24.8)	892 (55.0)	328 (20.2)	1.00	-	1.00	-
Colon cancer	295 (22.1)	764 (57.3)	274 (20.6)	1.14 (0.98-1.32)	0.108	0.98 (0.83-1.14)	0.779
Women[1]	145 (21.3)	389 (57.2)	146 (21.5)	1.16 (0.94-1.45)	0.176	0.98 (0.79-1.22)	0.912
Men[2]	150 (23.0)	375 (57.4)	128 (19.6)	1.11 (0.89-1.37)	0.387	0.96 (0.77-1.21)	0.772
Left Colon cancer	117 (21.27)	326 (59.27)	107 (19.5)	1.19 (0.96-1.49)	0.117	0.91 (0.72-1.14)	0.427
Women[1]	55 (20.8)	158 (59.6)	52 (19.6)	1.21 (0.88-1.66)	0.274	0.88 (0.63-1.22)	0.468
Men[2]	62 (21.8)	168 (58.9)	55 (19.3)	1.19 (0.88-1.61)	0.295	0.94 (0.69-1.30)	0.749
Lung cancer	263 (23.1)	620 (54.4)	257 (22.5)	1.08 (0.92-1.26)	0.376	1.10 (0.93-1.29)	0.275
Women[1]	88 (20.6)	245 (57.4)	94 (22)	1.22 (0.94-1.58)	0.156	1.02 (0.79-1.32)	0.895
Men[2]	175 (24.5)	375 (52.6)	163 (22.9)	1.01 (0.83-1.24)	0.917	1.17 (0.95-1.45)	0.152
Breast cancer[1]	332 (22.8)	804 (55.1)	322 (22.1)	1.07 (0.91-1.27)	0.442	1.02 (0.86-1.21)	0.827
Prostate cancer[2]	494 (23.1)	1225 (57.2)	422 (19.7)	1.10 (0.95-1.28)	0.231	0.97 (0.82-1.14)	0.711

Table S3 (B)

SNP309 TG

	Genotype *n* (%)			OR (95% CI)	*p*-value	OR (95% CI)
	**CC**	**CT**	**TT**	**CT+TT vs CC**		**TT vs CC+CT**
Controls	631 (35.6)	1143 (64.4)	1 (0.06)	1.00	-	1.00
Women[1]	314 (35.9)	561 (64.1)	0 (0)	1.00	-	1.00
Men[2]	317 (35.2)	582 (64.7)	1 (0.1)	1.00	-	1.00
Colon cancer	222 (31.4)	484 (68.4)	2 (0.3)	1.21 (1.00-1.46)	0.049	na
Women[1]	110 (30.5)	250 (69.3)	1 (0.3)	1.28 (0.98-1.66)	0.075	na
Men[2]	112 (32.3)	234 (67.4)	1 (0.3)	1.14 (0.88-1.49)	0.352	na
Left Colon cancer	93 (31.1)	205 (68.6)	1 (0.3)	1.24 (0.95-1.61)	0.117	na
Women[1]	44 (29.7)	103 (69.6)	1 (0.7)	1.32 (0.91-1.93)	0.162	na
Men[2]	49 (32.5)	102 (67.5)	0 (0)	1.13 (0.78-1.63)	0.58	na
Lung cancer	201 (34.8)	376 (65.1)	1 (0.2)	1.04 (0.85-1.26)	0.764	na
Women[1]	65 (32.5)	135 (67.5)	0 (0)	1.16 (0.84 -1.61)	0.412	na
Men[2]	136 (36.0)	241 (63.8)	1 (0.3)	0.97 (0.75-1.24)	0.798	na
Breast cancer[1]	266 (33.7)	523 (66.3)	0 (0)	1.10 (0.899-1.35)	0.354	na
Prostate cancer[2]	386 (33.2)	775 (66.8)	0 (0)	1.09 (0.91-1.31)	0.349	na

Table S3 (C)

SNP309 TT

	Genotype *n* (%)			OR (95% CI)	*p*-value	OR (95% CI)	*p*-value
	CC	CT	TT	CT+TT vs CC		TT vs CC+CT	
Controls	156 (10.7)	620 (42.7)	676 (46.5)	1.00	-	1.00	-
Women[1]	71 (9.7)	310 (42.5)	349 (47.8)	1.00	-	1.00	-
Men[2]	85 (11.8)	310 (42.9)	327 (45.3)	1.00	-	1.00	-
Colon cancer	73 (11.7)	280 (44.8)	272 (43.5)	0.91 (0.68-1.22)	0.542	0.89 (0.732-1.07)	0.212
Women[1]	35 (10.97)	139 (43.57)	145 (45.5)	0.87 (0.57-1.34)	0.578	0.91 (0.7-1.19)	0.502
Men[2]	38 (12.4)	141 (46.1)	127 (41.5)	0.94 (0.63-1.42)	0.754	0.86 (0.65-1.12)	0.272
Left Colon cancer	24 (9.6)	121 (48.2)	106 (42.2)	1.14 (0.72-1.79)	0.657	0.84 (0.64-1.10)	0.217
Women[1]	11 (9.4)	55 (47.0)	51 (43.6)	1.04 (0.53-2.02)	1	0.84 (0.57-1.25)	0.426
Men[2]	13 (9.7)	66 (49.3)	55 (41.0)	1.24 (0.67-2.3)	0.557	0.84 (0.58-1.22)	0.395
Lung cancer	62 (11.0)	244 (43.4)	256 (45.6)	0.97 (0.71-1.33)	0.873	0.96 (0.79-1.17)	0.691
Women[1]	23 (10.1)	110 (48.5)	94 (41.4)	0.96 (0.58-1.57)	0.898	0.77 (0.57-1.04)	0.094
Men[2]	39 (11.6)	134 (40)	162 (48.4)	1.01 (0.68-1.52)	1	1.13 (0.58-1.22)	0.354
Breast cancer[1]	66 (9.9)	281 (42.0)	322 (48.1)	0.98 (0.69- 1.40)	0.929	1.01 (0.82-1.25)	0.915
Prostate cancer[2]	108 (11.0)	450 (45.9)	422 (43.1)	1.08 (0.8-1.46)	0.643	0.91 (0.58-1.23)	0.374

[1] calculations against female healthy controls.

[2] calculations against male healthy controls.

Supplementary Table S4. Effect of MDM2 SNP55, SNP285, and SNP309 haplotypes on colon, lung, breast, and prostate cancers.

	CGG	TGT	total	CGG%	TGT%	Total%	OR	CI	*p* value
healthy control	2,516	3,116	5,632	44.7	55.3	100	1.00	-	-
women	1,259	1,569	2,828	44.5	55.5	100	1.00	-	-
men	1,257	1,547	2,804	44.8	55.2	100	1.00	-	-
Colon cancer	967	1,310	2,277	42.5	57.5	100	1.09	0.99-1.21	0.076
women	484	680	1,164	41.6	58.4	100	1.13	0.98- 1.29	0.092
men	483	630	1,113	43.4	56.6	100	1.06	0.92- 1.22	0.433
Lung cancer	841	1,133	1,974	42.6	57.4	100	1.09	0.98-1.21	0.114
Breast cancer	1,183	1,448	2,631	45.0	55.0	100	0.99	0.90-1.08	0.794
Prostate cancer	1,653	2,069	3,722	44.4	55.6	100	1.01	0.93-1.1	0.832

	CGG	CGT	total	CGG%	CGT%	Total%	OR	CI	*p* value
healthy control	2,516	1,563	4,079	61.7	38.3	100	1.00	-	-
women	1,259	766	2,025	62.2	37.8	100	1.00	-	-
men	1,257	797	2,054	61.2	38.8	100	1.00	-	-
Colon cancer	967	648	1615	59.9	40.1	100	1.08	0.96-1.21	0.216
women	484	319	803	60.3	39.7	100	1.08	0.92 -1.28	0.368
men	483	329	812	59.5	40.5	100	1.07	0.91 -1.27	0.396
Lung cancer	841	569	1,410	59.7	40.4	100	1.09	0.96- 1.23	0.183
Breast cancer	1,183	679	1,862	63.5	36.5	100	0.92	0.83- 1.04	0.175
Prostate cancer	1,653	1,052	2,705	61.1	38.9	100	1.02	0.93- 1.13	0.647

	CGG	CCG	total	CGG%	CCG%	Total%	OR	CI	*p* value
healthy control	2,516	254	2,770	90.8	9.2	100	1.00	-	-
women	1,259	122	1,381	91.2	8.8	100	1.00	-	-
men	1,257	132	1,389	90.5	9.5	100	1.00	-	-
Colon cancer	967	103	1,070	90.4	9.6	100	1.06	0.83 -1.34	0.665
women	484	60	544	89.0	11.0	100	1.28	0.92 -1.77	0.142
men	483	43	526	91.8	8.2	100	0.85	0.59-1.22	0.424
Lung cancer	841	98	939	89.6	10.4	100	1.15	0.90- 1.48	0.273
Breast cancer	1,183	104	1,287	91.9	8.1	100	0.87	0.69- 1.11	0.284
Prostate cancer	1,653	188	1,841	89.8	10.2	100	1.13	0.92- 1.37	0.241

	TGT	CGT	total	TGT%	CGT%	Total%	OR	CI	*p* value
healthy control	3,116	1,563	4,679	66.6	33.4	100	1.00	-	-
women	1,569	766	5,335	29.4	70.6	100	1.00	-	-
men	1,547	797	2344	66.00	34.0	100	1.00	-	-
Colon cancer	1308	648	1956	66.9	33.1	100	0. 99	0.88-1.11	0.842
women	680	319	999	68.1	31.9	100	0.96	0.82-1.13	0.629
men	630	329	959	65.7	34.3	100	1.01	0.87-1.19	0.872
Lung cancer	1132	569	1701	66.6	33.5	100	1.00	0.89-1.13	0.976
Breast cancer	1448	679	2127	68.1	31.9	100	0.94	0.84-1.04	0.232
Prostate cancer	2069	1,052	3121	66.3	33.7	100	1.014	0.92-1.12	0.788

	TGT	CCG	total	TGT%	CGG%	Total%	OR	CI	*p* value
healthy control	3,116	254	3370	92.5	7.5	100	1.00	-	-
women	1,569	122	1691	92.8	7.2	100	1.00	-	-
men	1,547	132	1679	92.1	7.9	100	1.00	-	-
Colon cancer	1308	103	1411	92.7	7.3	100	0.97	0.76-1.23	0.810
women	680	60	740	91.9	8.1	100	1.14	0.82-1.57	0.451
men	630	43	673	93.6	6.4	100	0.80	0.56-1.14	0.258
Lung cancer	1132	98	1230	92.0	8.7	100	1.06	0.83-1.35	0.617
Breast cancer	1448	104	1552	93.3	6.7	100	0.88	0.70-1.12	0.315
Prostate cancer	2069	188	2257	91.7	8.3	100	1.12	0.92-1.36	0.289

	CGT	CCG	total	%	%	Total%	OR	CI	*p* value
healthy control	1,563	254	1,817	86.0	14.0	100	1.00	-	-
women	766	122	888	86.3	13.7	100	1.00	-	-
men	797	132	929	85.8	14.2	100	1.00	-	-
Colon cancer	648	103	751	86.3	13.7	100	0.98	0.76-1.25	0.900
women	319	60	379	84.2	15.8	100	1.18	0.84-1.65	0.337
men	329	43	372	88.4	11.6	100	0.79	0.55-1.14	0.242
Lung cancer	569	98	667	85.3	14.7	100	1.06	0.82-1.36	0.650
Breast cancer	679	104	783	86.7	13.3	100	0.94	0.74-1.21	0.664
Prostate cancer	1,052	188	1,240	84.8	15.2	100	1.10	0.90-1.35	0.373

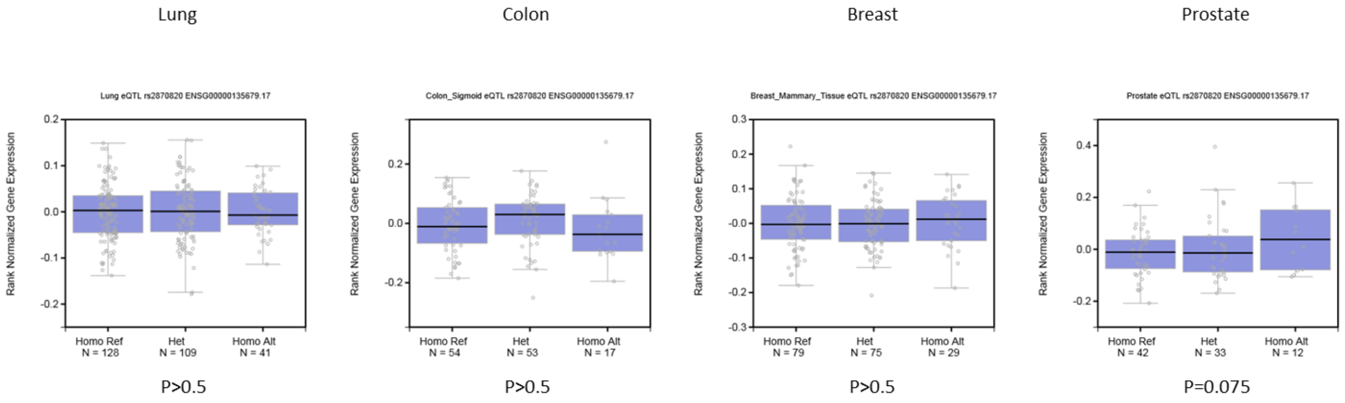

Data extracted from www.gtexportal.org

Supplementary Figure S1. Expresssion levels of MDM2 in lung-, colon-, breast- and prostate tissue, stratified according to MDM2SNP55 genotype.
